# Combination of the gamma-glutamyltransferase-to-prealbumin ratio and other indicators may be a novel marker for predicting the prognosis of patients with hepatocellular carcinoma undergoing locoregional ablative therapies

**DOI:** 10.1186/s13027-019-0266-1

**Published:** 2019-12-19

**Authors:** Q. Wang, P. Zhao, N. He, J. P. Sun, K. Li, C. R. Zang, Y. N. Zhao, Y. Zhao, Y. H. Zhang

**Affiliations:** 1 Research center for biomedical resources, Beijing You’an Hospital, Capital Medical University, Beijing, 100069 China; 2 Interventional therapy center for oncology, Beijing You’an Hospital, Capital Medical University, Beijing, 100069 China; 3 Clinical detection center, Beijing You’an Hospital, Capital Medical University, 8 Xitoutiao, Youanmenwai Street, Fengtai District, Beijing, 100069 China; 4 Research center for biomedical resources; Interventional therapy center for oncology; Beijing You’an Hospital, Capital Medical University, 8 Xitoutiao, Youanmenwai Street, Fengtai District, Beijing, China

**Keywords:** Gamma-glutamyltransferase, Prealbumin, Hepatocellular carcinoma, Transcatheter arterial chemoembolization, Locoregional ablation, Prognosis

## Abstract

**Objective:**

The aim of this study was to investigate the prognostic significance of the serum γ-glutamyltransferase (γ-GT)-to-prealbumin ratio (GPR) and whether combining this ratio with other parameters can lead to an improved prognostic value for patients with hepatocellular carcinoma (HCC) undergoing transcatheter arterial chemoembolization (TACE) combined with local ablation therapy.

**Methods:**

A total of 235 HCC patients who were treated with combined therapies were retrospectively analyzed. The demographic data and clinicopathological data were collected. A fibrinogen (Fib)-GPR score of 2 was assigned to patients with elevated Fib and GPR values, and a score of 1 or 0 was assigned to patients with one or neither of these two markers, respectively. In addition, an N-score of 2 was assigned to patients with low neutrophil and high GPR values, and a score of 1 or 0 was assigned to patients with one or neither of these two markers, respectively. The optimal cutoff values and prognostic roles of GPR and other markers were identified according to the time-dependent receiver operating characteristic (ROC) curves and Youden’s index.

**Results:**

Multiple tumors, high levels of α-fetoprotein (AFP) and Fib, as well as a high GPR, were found to be independent risk factors in recurrent patients, while multiple tumors, a low neutrophil count, and a high GPR were associated with reduced overall survival (OS) in patients with HCC who received combined therapies. Patients with a Fib-GPR score of 2 and N-GPR score of 2 had poor recurrence-free survival (RFS) and OS, respectively.

**Conclusions:**

Fib-GPR and N-GPR scores may be helpful in predicting both recurrence and the prognosis of HCC patients, thereby assisting in the process to make a true clinical decision and optimize therapeutic options.

## Strengths and limitations of this study

1. The proposed Fib-GPR and N-GPR scores for HCC patients might effectively predict recurrence and mortality.

2. Our scores, which were based on γ-GT, may possess some advantages for making a true clinical decision for patients with HCC undergoing TACE plus local ablation therapy.

3. This is a retrospective single-center study and thus is affected by confounding factors; therefore, our results need to be validated by further multicenter studies.

## Background

Hepatocellular carcinoma (HCC) is the third leading cause of cancer deaths and the sixth most common cancer worldwide, with approximately 841,000 new cases and 782,000 deaths annually [1]. In China, HCC accounts for more than 83.9~92.3% of primary liver cancer cases and is currently a serious health problem [2, 3]. In recent years, significant advances in locoregional therapies have led to excellent results with therapy that are highly comparable to those with surgical resection, especially in patients with small single tumors because the therapy are associated with minimal invasion, rapid recovery, and few complications. Locoregional ablation plus transcatheter arterial chemoembolization (TACE), which can reduce the tumor bulk and heat sink effect, are potential treatment strategies for patients who are not eligible for surgical resection due to dysfunction or coagulopathy [4]. However, high recurrence and low overall survival (OS) rates reduce the quality of life of HCC patients and thus should be urgently managed. Therefore, valid markers for predicting patients’ prognosis are of great significance in choosing the optimal therapeutic modality.

As an almost ubiquitous enzyme, γ-glutamyltransferase (γ-GT) initiates the degradation of extracellular glutathione and is also conjugates and correlates with biotransformation, nucleic acid metabolism, and oncogenesis [5]. Previous studies have shown that high levels of γ-GT are significantly associated with unfavorable prognoses in HCC patients [6, 7]. Prealbumin (PA), which is synthesized by the liver, is a recently identified biomarker indicative of liver synthetic functions and has not been included in the traditional Child-Pugh classification system. Several scholars have demonstrated that low preoperative serum levels of PA could be used to predict the long-term prognosis of HCC patients undergoing liver resection [8, 9].

A limited number of studies have investigated whether γ-GT combined with PA could predict the prognosis of HCC patients treated with TACE plus locoregional ablation. Therefore, the present research aimed to study the data of HCC patients who received combined therapies as the primary treatment and validate the predictive value of combining GPR with other parameters for HCC patients.

## Methods

### Study subjects

A retrospective analysis of 235 patients with HCC who received combined therapies as initial treatment was conducted in Beijing You’an Hospital (Beijing, China) between January 1, 2016 and December 31, 2017. HCC was diagnosed according to the radiological/histological criteria recommended by the guidelines published by the American Association for the Study of Liver Diseases (AASLD) [10]. All subjects were required to meet the following inclusion criteria: 1) age in the range of 18–75 years; 2) TACE plus ablative therapy was the initial anticancer treatment; 3) Child-Pugh class A or B; and 4) no other malignancies that may affect the prognosis. The exclusion criteria were as follows: 1) radiological evidence of invasion into the major portal/hepatic vein branches; 2) presence of extrahepatic metastases; 3) severe coagulation disorders; 4) incomplete ablation; 5) secondary liver cancer; and 6) missed follow-up examinations.

Standard demographic and clinicopathological data were collected and summarized as follows: 1) demographic data, such as age, sex, history of hypertension, and diabetes mellitus; 2) etiologies of HCC, including hepatitis B virus (HBV), hepatitis C virus (HCV), and alcoholic liver disease (ALD); 3) tumor-related indices, such as the number of tumors, size of tumors, and alpha-fetoprotein (AFP) level; 4) liver function indices, including cirrhosis, Child-Pugh class, and alanine aminotransferase (ALT), aspartate aminotransferase (AST), total bilirubin, serum albumin, globulin, and γ-GT levels; 5) routine blood examinations, such as neutrophil count, lymphocyte count, platelet count, and Fib and GPR level; and 6) ablation modalities, including radiofrequency ablation (RFA), microwave ablation (MWA), and argon-helium cryoablation (AHC). This study was conducted in accordance with the 1964 Declaration of Helsinki, and the study protocol was approved by the Ethics Committee of Beijing You’an Hospital; the patient data were kept confidential. As a study with minimum risk, the requirement for informed consent was waived because it was arduous to contact the patients again.

### TACE procedure

The procedure was performed by two qualified hepatologists. With the selective/superselective technique, the tumor-feeding arteries were catheterized using a highly flexible coaxial microcatheter passed through a 5-Fr Yashiro catheter (Terumo, Tokyo, Japan), which was previously placed approximately in the hepatic artery. After placing the microcatheter, a mixture of doxorubicin (Pfizer Inc., New York, NY, USA) and lipiodol (Guerbet, Villepinte, France) was injected; then, embolization was performed with embolic materials, such as gelfoam or polyvinyl alcohol particles, until full stasis was achieved in the segmental or subsegmental arterial branches in the tumor-feeding vessels. The dose of the drugs depended on the patient’s white blood cell count, platelet count, and liver function. Angiography showed occlusions in the tumor vasculature, filling of the embolic agent, and disappearance of the tumor stain, which was regarded as the endpoint of embolization.

### Ablation procedure

Thermal ablation was carried out within 2 weeks after TACE. The procedure was percutaneously conducted under local anesthesia by qualified hepatologists with the guidance of triple-phase computed tomography (CT) or magnetic resonance imaging (MRI). The procedure was as follows: 1) the ablation procedure was determined according to the CT or MRI scans with the patient in the proper position; 2) the area was disinfected, towels were laid down, anesthesia was administered at the puncture site, and the ablation needle was inserted into the skin; 3) to perform the ablation, multiple sites and overlapping ablation were considered according to the number of tumors and size of the tumors, and CT scans were acquired in a timely manner to follow the ablation process; and 4) after the ablation was completed, the ablation needle was pulled out, and the needle tract was ablated to prevent needle transplantation and bleeding. Regardless of how many ablation sessions were performed, the range of ablation extended 0.5–1.0 cm into the surrounding noncancerous tissue to ensure full coverage; otherwise, the procedure was defined as an incomplete ablation.

### Follow-up

All patients were followed-up in the outpatient clinic. Abdominal CT or MRI was carried out 4–6 weeks after treatment. The follow-up involved a physical examination and blood tests, including assessments of liver function and AFP levels, as well as medical imaging examinations that included an abdominal ultrasound every 3–6 months and triple-phase CT/MRI every 6 months. Recurrence included local recurrence, distant intrahepatic recurrence, and extrahepatic metastasis [11]. Recurrence-free survival (RFS) was defined as the time between the date that ablation was terminated and the first instance of detectable recurrence or the mortality date of patients without evidence of disease recurrence, while OS was calculated as the time between the date that ablation was terminated and the date of tumor-related mortality or the last follow-up date; in the present study, the closing date was April 1, 2019. When recurrence was recognized, patients were treated by RFA or TACE. Recurrence was diagnosed based on enhanced CT or MRI examinations. If the imaging examination showed an enhanced area within or around the original tumor, recurrence was considered.

### Patient and public involvement

Anonymized patient data were used in the present study. Patients and the public were not involved in the study process.

### Statistical analysis

All data were analyzed with SPSS 25.0 software (IBM, Armonk, NY, USA). Continuous variables were expressed as the mean ± standard deviation (SD), and categorical data were presented as the frequency. The baseline GPR data were compared with the Mann-Whitney U test. Univariate and multivariate Cox regression analyses were performed to assess the independent risk factors of prognosis in HCC patients undergoing combined therapies. The RFS and OS rates were calculated with the Kaplan-Meier method, and the differences between groups were compared by using the log-rank test. The optimal cutoff values were identified according to the receiver operating characteristic (ROC) curves and Youden’s index. All statistical tests were two-sided, and a *P-value* < 0.05 was considered statistically significant.

## Results

### Clinical characteristics of HCC patients

This cohort consisted of 183 males (77.9%) and 52 females (22.1%) with a mean age of 58 ± 8 years (range, 27~74 years). Additionally, 64 patients (27.2%) had high blood pressure, and 50 (21.3%) patients had type 2 diabetes mellitus. In addition, 124 patients (52.8%) were treated with antiviral therapy before they underwent combined therapies. There were 98 patients (41.7%) who had a history of smoking and 76 patients (32.3%) with a history of drinking. In terms of etiologies, 177 (75.3%) patients had HBV-related HCC, 43 (18.3%) patients had HCV-related HCC, and 15 (6.4%) patients had ALD-related HCC. The median follow-up duration was 38.2 months (25~75th percentiles, 36.0~43.3 months). The 1-, 2-, and 3-year cumulative recurrence rates were 24.3% (57/235), 48.1% (113/235), and 59.1% (139/235), respectively. Moreover, the 1-, 2-, and 3-year cumulative OS rates were 98.3% (231/235), 94.0% (221/235), and 88.5% (208/235), respectively (Table 1).

**Table 1:** Demographic and clinicopathological data in HCC patients

Variables	Mean ± SD/n (%)
Gender, male/female (%)	183 (77.9%)/52 (22.1%)
Age,≤60 years/>60 years(%)	143 (60.9)/92 (39.1)
Diabetes mellitus (%)	50 (21.3%)
High blood pressure (%)	64 (27.2%)
History of smoking (%)	98 (41.7%)
History of drinking (%)	76 (32.3%)
Cirrhosis (%)	180 (76.6%)
Child-Pugh class, A/B (%)	154 (65.5%)/81 (34.5%)
Antiviral therapy (%)	124 (52.8%)
Tumor number, solitary/multiple (%)	167 (71.1%)/68 (28.9%)
Tumor size, <30 mm vs ≥30 mm	154 (65.5%)/81 (34.5%)
Ablation method, RFA/MWA/AHC (%)	117 (49.8%)/61 (26%)/57 (24.3%)
Etiology, HBV/HCV/ALD (%)	177 (75.3%)/43 (18.3%)/15 (6.4%)
AFP (< 7 ng/mL/7-400 ng/mL/≥400 ng/mL)	107 (45.5%)/99 (42.1%)29 (12.4%)
Viral load(< 100 IU/mL/≥100 IU/mL)	139 (59.1%)/96 (40.9%)
ALT(U/L)	37.82 ± 20.40
AST(U/L)	32.59 ± 12.62
Total serum bilirubin (mg/dL)	19.16 ± 9.46
Neutrophil count (10^9/L)	3.26 ± 1.64
Lymphocyte count (10^9/L)	1.22 ± 0.57
Platelet count (10^9/L)	125.15 ± 64.62
Albumin(g/L)	36.89 ± 4.68
Globulin(g/L)	27.92 ± 6.50
Fibrinogen (mg/dL)	2.93 ± 0.93
GPR	0.70 ± 1.01

Abbreviations: *RFA* radiofrequency ablation, *MWA* microwave ablation, *AHC* argon-helium cryoablation, *HBV* hepatitis B virus, *HCV* hepatitis C virus, *ALD* alcohol liver disease, *AFP* alpha-fetoprotein, *ALT* alanine aminotransferase, *AST* aspartate aminotransferase, *GPR* gamma-glutamyltransferase to prealbumin ratio, the GPR was estimated as the gamma-glutamyltransferase divided by the prealbumin

### Prognostic factors associated with RFS

Univariate and multivariate analyses were performed to assess the associations between clinical characteristics and RFS. The univariate analysis revealed that RFS was significantly associated with the significant factors shown in Table 2, as well as the albumin, globulin, and Fib levels and GPR. The multivariate analysis showed that the number of tumors (HR: 1.98; 95%CI: 1.38–2.85), AFP level (HR: 1.29; 95%CI: 1.01–1.66), Fib level (HR: 1.23; 95%CI: 1.00–1.50), and GPR (HR: 1.15; 95%CI: 1.00–1.33) were independent predictors of HCC recurrence (Table 2).

**Table 2:** Prognostic factors for RFS by Cox proportional hazards regression model

Factors	Univariate		Multivariate	
	HR (95% CI)	P	HR (95% CI)	P
Gender	1.72 (1.10–2.69)	0.016	1.63 (0.97–2.72)	0.061
Age	0.88 (0.62–1.23)	0.466		
Diabetes mellitus	1.37 (0.93–2.01)	0.108		
High blood pressure	1.03 (0.71–1.49)	0.866		
History of smoking	1.51 (1.08–2.09)	0.014	1.11 (0.76–1.63)	0.579
History of drinking	1.22 (0.86–1.72)	0.248		
Cirrhosis	1.28 (0.85–1.92)	0.222		
Child-Pugh class	1.27 (0.90–1.78)	0.166		
Antiviral therapy	0.77 (0.55–1.08)	0.135		
Tumor number	2.36 (1.68–3.32)	< 0.0001	1.98 (1.38–2.85)	< 0.0001
Tumor size	1.60 (1.15–2.24)	0.005	0.93 (0.61–1.41)	0.748
Ablation method	0.90 (0.74–1.10)	0.341		
Etiology	1.71 (0.89–1.53)	0.250		
AFP	1.44 (1.13–1.82)	0.002	1.29 (1.01–1.66)	0.041
Viral load	1.00 (0.75–1.31)	0.291		
ALT(U/L)	1.00 (0.99–1.01)	0.237		
AST(U/L)	1.01 (0.99–1.02)	0.062		
Total serum bilirubin (mg/dL)	1.00 (0.99–1.02)	0.369		
Neutrophil count (10^9/L)	1.08 (0.98–1.19)	0.113		
Lymphocyte count (10^9/L)	0.99 (0.75–1.31)	0.970		
Platelet count (10^9/L)	1.00 (0.99–1.00)	0.147		
Albumin	0.95 (0.91–0.98)	0.012	0.97 (0.92–1.01)	0.193
Globulin	1.03 (1.00–1.06)	0.015	1.01 (0.99–1.05)	0.206
Fibrinogen (mg/dL)	1.32 (1.12–1.55)	0.001	1.23 (1.00–1.50)	0.042
GPR	1.27 (1.13–1.41)	< 0.0001	1.15 (1.00–1.33)	0.048

Abbreviations: *AFP* alpha-fetoprotein, *ALT* alanine aminotransferase, *AST* aspartate aminotransferase, *GPR* gamma-glutamyltransferase to prealbumin ratio, the GPR was estimated as the gamma-glutamyltransferase divided by the prealbumin

### Analysis of the effect of different fib levels, GPR values, and fib-GPR values on RFS

**Fig. 1:**
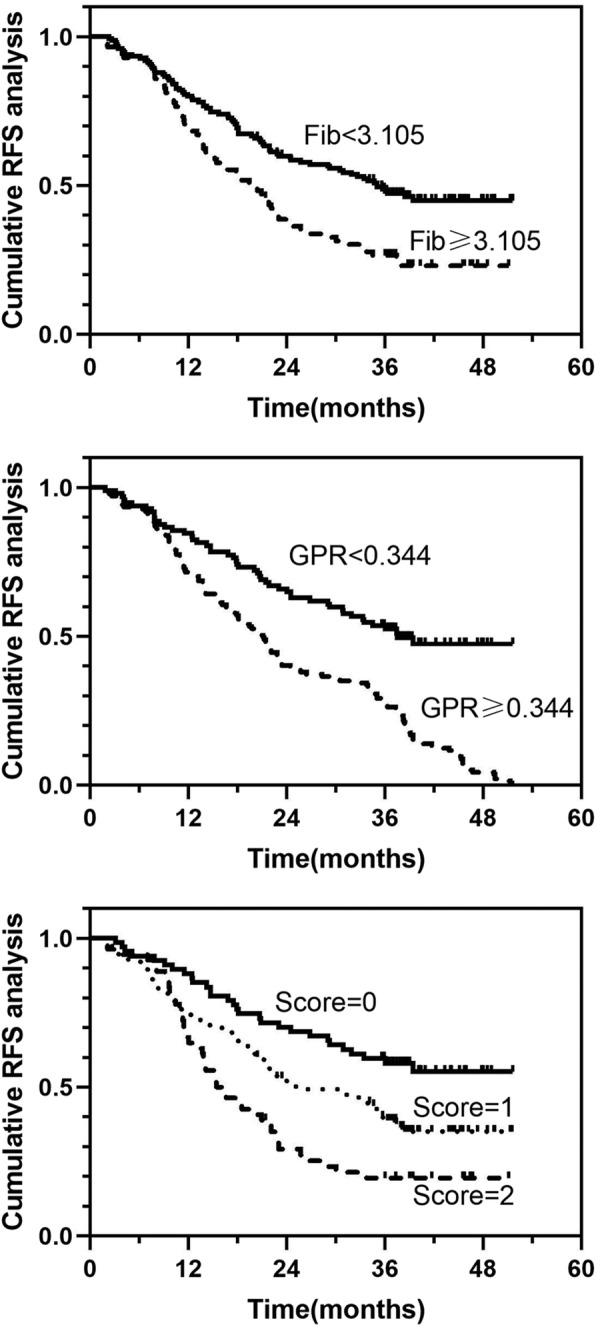
Kaplan-Meier analysis of RFS for patients with different levels of fibrinogen, GPR, and Fib-GPR scores. Abbreviations: Fib: fibrinogen; GPR: gamma-glutamyltransferase to prealbumin ratio, the GPR was estimated as the gamma-glutamyltransferase divided by the prealbumin

**Fig. 2:**
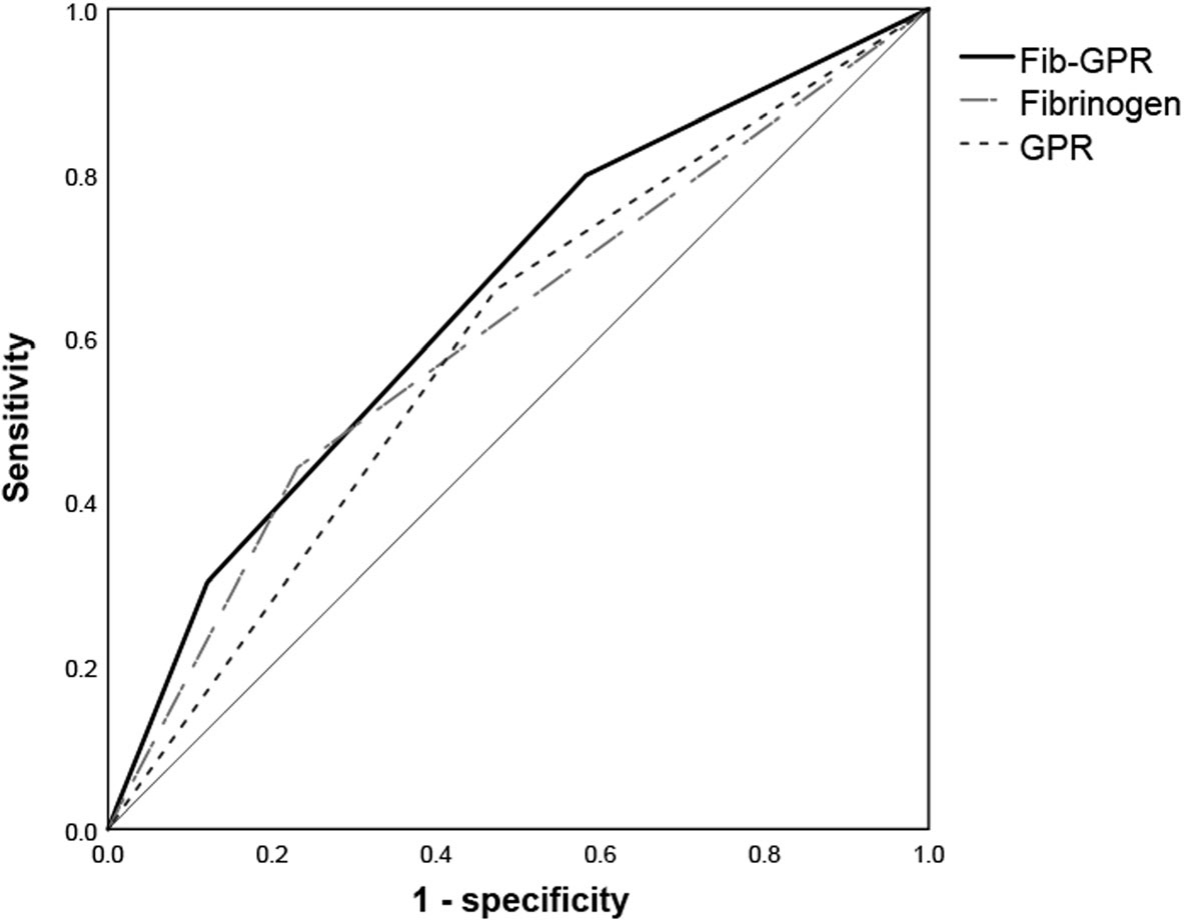
ROC curves for fibrinogen, GPR, and Fib-GPR scores. Abbreviations: GPR: gamma-glutamyltransferase to prealbumin ratio, the GPR was estimated as the gamma-glutamyltransferase divided by the prealbumin

Based on the time-dependent ROC curves and Youden’s index, the cutoff values for the Fib level and GPR were 3.105 g/L and 0.344, respectively. To explore whether the combination of the Fib level and GPR could lead to an improved predictive value, the patients were divided into three groups according to three different scores: score 0 (Fib level < 3.105 g/L and GPR < 0.344), score 1 (Fib level < 3.105 g/L or GPR < 0.344), and score 2 (Fib level ≥ 3.105 g/L and GPR ≥ 0.344) (Table 3). The Kaplan-Meier analysis confirmed that the Fib level, GPR, and Fib-GPR score were positive predictors for patients with HCC. There were statistically significant differences in RFS among different Fib and GPR levels and Fib-GPR scores for patients who underwent combined therapies (*P* < 0.0001, *P* = 0.002, and *P* < 0.001, respectively) (Fig. 1). The time-dependent ROC curves and areas under the curves (AUCs) showed that the Fib-GPR score (0.647), which was used to predict the 2-year RFS rate, was superior to Fib (0.605) and GPR (0.592) levels alone (Fig. 2). The median RFS duration was 36.2, 25.7, and 15.8 months in patients with scores of 0, 1, and 2, respectively. The cumulative 1-, 2-, and 3-year RFS rates in patients with a score of 0 after combined treatments were 11.9, 29.9, and 41.8%, while those in patients with scores of 1 and 2 were 25.7, 47.8, 59.3, and 35.2%, 70.4, 79.6%, respectively.

**Table 3:** Two scoring system for RFS and OS

Score	Fib-GPR scores	N-GPR scores
0	Fib< 3.105 g/L and GPR < 0.344	*N* ≥ 4.145 × 10^9/L and GPR < 0.602
1	Fib< 3.105 g/L or GPR < 0.344	N ≥ 4.145 × 10^9/L or GPR < 0.602
2	Fib≥3.105 g/L and GPR ≥ 0.344	*N* < 4.145 × 10^9/L and GPR ≥ 0.602

Abbreviations: *Fib* fibrinogen, *N* neutrophil

### Prognostic factors associated with the OS

Univariate and multivariate analyses were used to evaluate the association between clinical characteristics and OS. The univariate analysis indicated that OS was significantly associated with factors such as the Child-Pugh class, neutrophil count, AST level, and total serum bilirubin. The multivariate analysis showed that the number of tumors (HR: 3.05; 95%CI: 1.32–7.02), neutrophil count (HR: 0.65; 95%CI: 0.47–0.91), and GPR (HR: 1.34; 95%CI: 1.00–1.81) were independent predictors of HCC recurrence (Table 4).

**Table 4:** Prognostic factors for OS by Cox proportional hazards regression model

Factors	Univariate		Multivariate	
	HR (95% CI)	P	HR (95% CI)	P
Gender	2.76 (0.84–9.07)	0.094		
Age	0.91 (0.44–1.87)	0.810		
Diabetes mellitus	1.49 (0.69–3.23)	0.307		
High blood pressure	1.51 (0.72–3.14)	0.269		
History of smoking	1.85 (0.92–3.73)	0.082		
History of drinking	1.25 (0.61–2.55)	0.541		
Cirrhosis	2.30 (0.80–6.56)	0.119		
Child-Pugh class	2.38 (1.18–4.78)	0.014	0.81 (0.29–2.21)	0.685
Antiviral therapy	0.67 (0.33–1.36)	0.273		
Tumor number	3.77 (1.87–7.60)	< 0.0001	3.05 (1.32–7.02)	0.009
Tumor size	2.24 (1.12–4.05)	0.022	2.05 (0.90–4.66)	0.084
Ablation method	1.36 (0.90–2.04)	0.137		
etiology	1.59 (0.97–2.60)	0.063		
AFP	1.65 (0.99–2.72)	0.050	1.03 (0.57–1.85)	0.914
viral load	1.22 (0.54–2.75)	0.624		
ALT(U/L)	1.00 (0.98–1.01)	0.773		
AST(U/L)	1.03 (1.00–1.05)	0.007	1.02 (0.99–1.05)	0.121
Total serum bilirubin (mg/dL)	1.03 (1.00–1.06)	0.040	1.02 (0.98–1.07)	0.262
Neutrophil count (10^9/L)	0.77 (0.60–0.99)	0.045	0.65 (0.47–0.91)	0.014
Lymphocyte count (10^9/L)	0.82 (0.43–1.57)	0.565		
Platelet count (10^9/L)	1.00 (0.99–1.01)	0.797		
Albumin	0.88 (0.81–0.95)	0.002	0.96 (0.86–1.06)	0.441
Globulin	1.05 (1.00–1.11)	0.023	0.99 (0.93–1.05)	0.841
Fibrinogen (mg/dL)	1.00 (0.69–1.46)	0.980		
GPR	1.34 (1.14–1.58)	< 0.0001	1.34 (1.00–1.81)	0.047

Abbreviations: *AFP* alpha-fetoprotein, *ALT* alanine aminotransferase, *AST* aspartate aminotransferase, *GPR* gamma-glutamyltransferase to prealbumin ratio, the GPR was estimated as the gamma-glutamyltransferase divided by the prealbumin

### Analysis of the effect of different neutrophil, GPR, and N-GPR values on OS

**Fig. 3:**
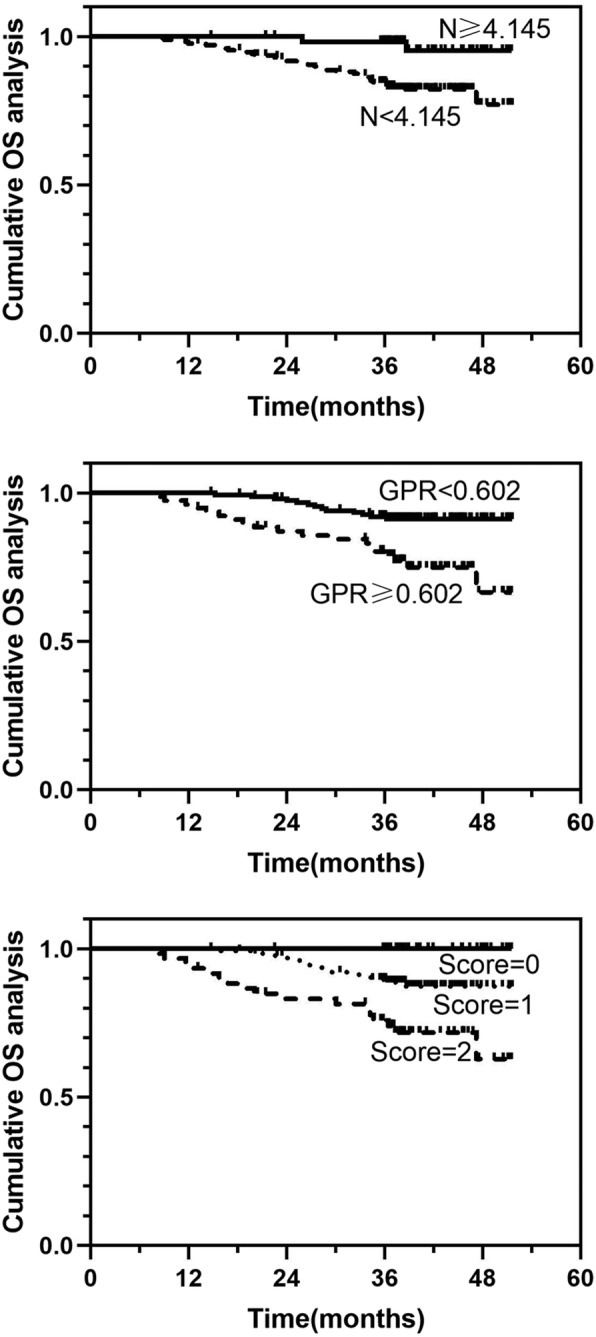
Kaplan-Meier analysis of OS for patients with different levels of neutrophil, GPR, and N-GPR scores. Abbreviations: N: neutrophil; GPR: gamma-glutamyltransferase to prealbumin ratio, the GPR was estimated as the gamma-glutamyltransferase divided by the prealbumin

**Fig. 4:**
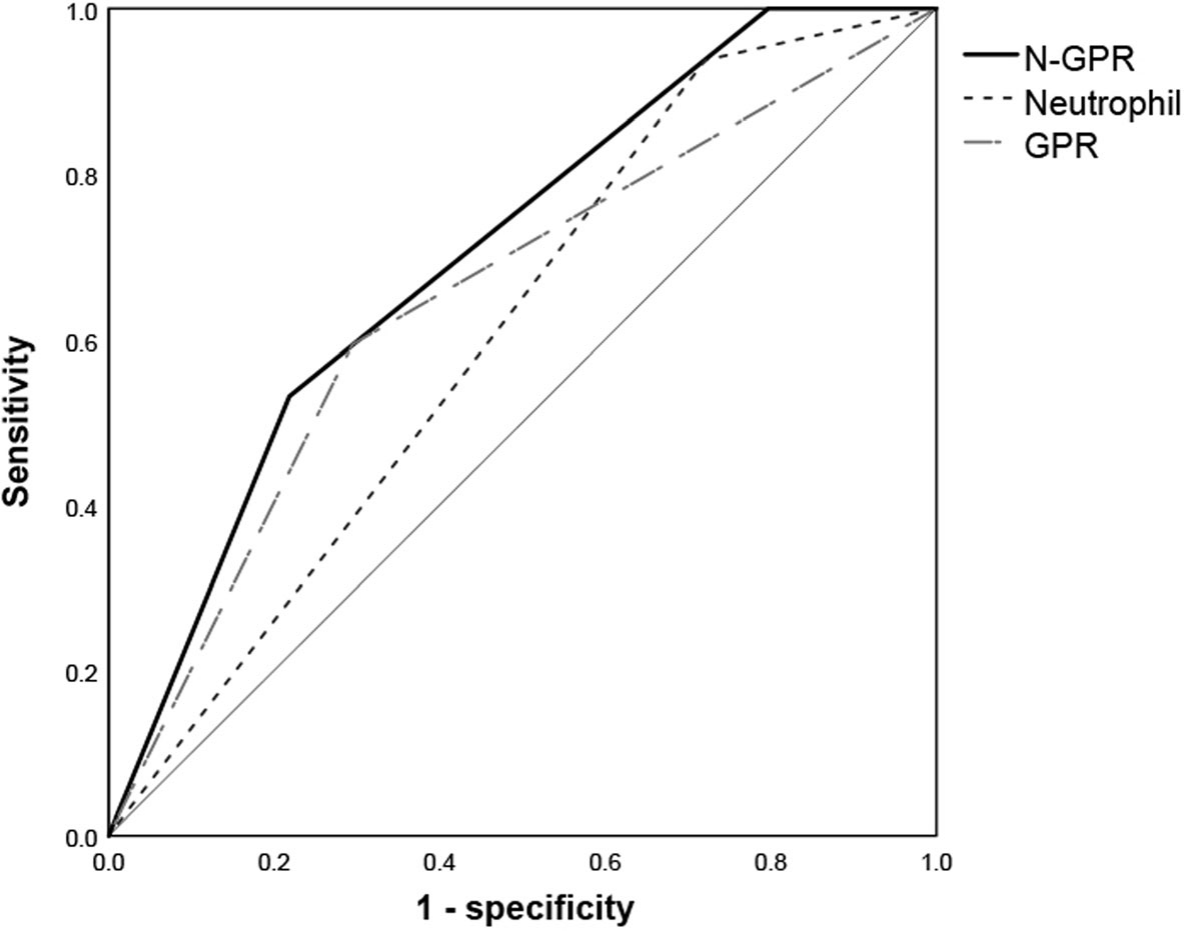
ROC curves for neutrophil, GPR, and N-GPR scores. Abbreviations: GPR: gamma-glutamyltransferase to prealbumin ratio, the GPR was estimated as the gamma-glutamyltransferase divided by the prealbumin

Based on the time-dependent ROC curves and Youden’s index, the cutoff values of the neutrophil count and GPR were 4.145 × 10^9/L and 0.602, respectively. The patients were divided into three groups according to three different scores to confirm whether combining the neutrophil count and GPR could lead to an improved predictive value: score 0 (neutrophil count≥4.145 × 10^9/L and GPR < 0.602), score 1 (neutrophil count≥4.145 × 10^9/L or GPR < 0.602), and score 2 (neutrophil count< 4.145 × 10^9/L and GPR ≥ 0.602) (Table 3). The Kaplan-Meier analysis revealed statistically significant differences in OS among different levels of neutrophil counts, GPR values, and N-GPR scores for patients who underwent combined therapies (*P* < 0.007, *P* = 0.001, and *P* < 0.001, respectively) (Fig. 3). The time-dependent ROC curves and the AUC values showed that the N-GPR score (0.704), which was used to predict the 2-year OS rate, was better than the neutrophil count (0.608) and GPR (0.650) alone (Fig. 4). The median OS duration were 39.4, 38.4, and 37.6 months in for patients with N-GPR scores of 0, 1, and 2, respectively. The cumulative 1-, 2-, and 3-year OS rates for patients with a score of 0 after combined treatments were 100, 100, and 100%, while those for patients with scores of 1 and 2 were 100, 96.9, 89.9, and 93.3%, 83.3, 75.0%, respectively.

### Correlations between GPR, tumor size and number of tumors

**Fig. 5:**
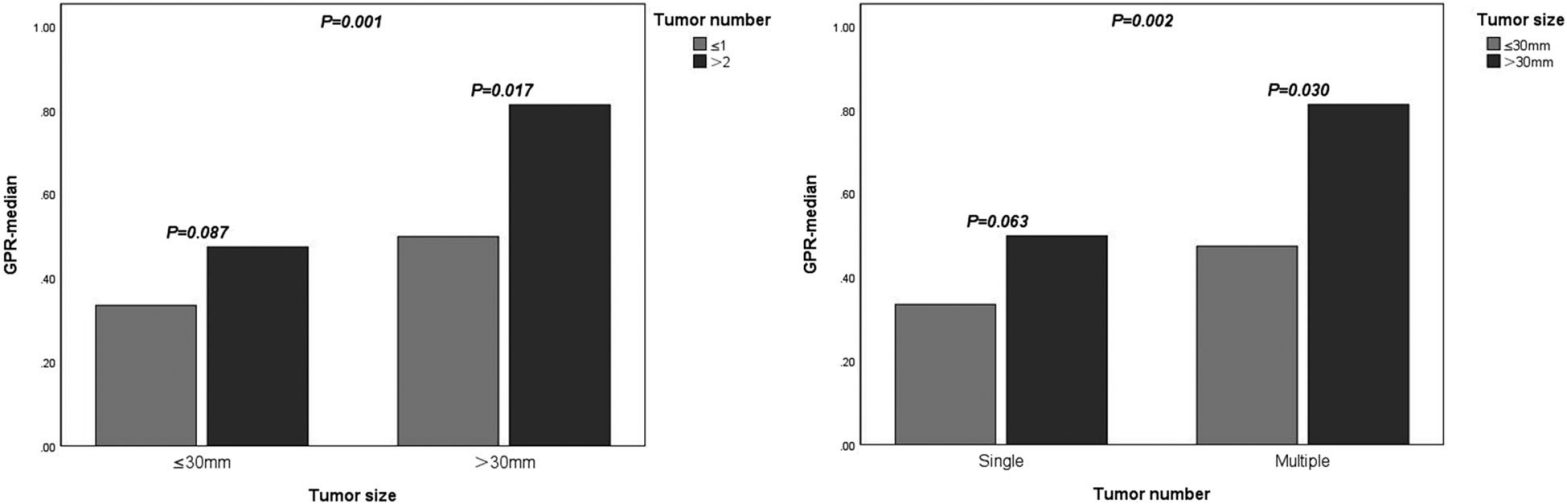
The correlation between GPR and the size and number of tumor

We further conducted Mann-Whitney U tests, and the results showed that GPR was higher in patients with large (> 30 mm) and multiple tumors than in patients with small (≤30 mm) and single tumors (*P* = 0.001 and 0.002, respectively). Furthermore, among the group with large tumors, the GPR was higher in patients with multiple tumors than in patients with a single tumor (*P* = 0.017), but this finding was not observed in the group with small tumors (*P* = 0.087). Additionally, among group with multiple tumors, the GPR was higher in patients with large tumors than in patients with small tumors (*P* = 0.030), but this finding was not observed in the group with single tumors (*P* = 0.063) (Fig. 5).

## Discussion

In China, the incidence and mortality of HCC globally accounts for approximately half of all patients with liver cancer. Hence, HCC has aggravated the medical burden in China and is a serious health problem [12]. TACE combined with ablation therapy is a potential therapeutic strategy for HCC; however, the problem of recurrence seriously influences the efficacy of combined treatments. Thus, an exploration for effective predictive indicators of RFS and OS is of great significance, and these predictors might assist clinicians in adopting timely strategies to prevent recurrence and improve outcomes of HCC patients.

The results of the present study showed that high Fib and AFP levels were independent risk factors for RFS in patients with HCC who underwent combined treatments. Elevated plasma Fib levels have been associated with tumor progression in several malignancies, including esophageal carcinoma, small cell lung carcinoma, and non-small cell lung carcinoma [13–17]. The following triggers might explain our findings. First, Fib may contribute to the adhesion of tumor cells to platelets, to platelet aggregation and thrombin formation around the tumor cells, thus protecting them against attacks from natural killer cells. Second, oncogenesis is typically accompanied by inflammatory responses and leukocyte infiltration into the tumor stroma, which may convert Fib to the fibrin matrix, thereby promoting tumor angiogenesis. Third, Fib may serve as an extracellular matrix that regulates the growth of cancer cells by binding to various types of growth factors, which may promote cellular adhesion, proliferation, and metastasis and inhibit the apoptosis of tumor cells [18, 19]. AFP, a biomarker for HCC, plays a substantial role in the regulation of hepatocarcinogenesis. However, the relationship of AFP with prognosis in patients with HCC remains elusive. The current research showed that a high AFP level is an independent risk factor for RFS in patients with HCC who received combined therapies, which was in agreement with Yang et al.’s conclusion [20]. The elevated AFP level may be an indication of vascular invasion and HCC progression, both of which contribute to an elevated risk for early recurrence [21].

The present study confirmed that low neutrophil levels are an independent risk factor for only OS in patients who received combined therapies, which is controversial with several results. For my part, liver disease may inhibit the hematopoietic function of marrow, another aspect is that hypersplenism may lead to neutropenia. However, bias resulting from fewer death cases cannot be ignored [22].

The multivariate regression analysis revealed that the number of tumors and GPR were independent predictors for both RFS and OS of HCC patients. Multiple tumors are typically characterized by the multicentric development of neoplasms. In addition, there may be microscopic lesions around the tumors that cannot be clearly detected by imaging examinations and are more likely to relapse and metastasize. A previous study reported that the expression of γ-GT provides tumor cells with an additional source of cysteine and cystine from the cleavage of extracellular glutathione and oxidized glutathione [23]. In addition, compared to that in patients with small tumors and single tumors, γ-GT levels are notably higher in individuals with large and multiple tumors, which are more likely to relapse [24]. Additionally, a number of previously conducted studies indicated that macrophages and neutrophils can facilitate the release of γ-GT, which disrupts the HCC microenvironment, causing the progression of cancer, and this finding was in agreement with our findings [25, 26]. PA is synthesized by the liver, and the half-life of PA is as short as 1.9 days, compared with albumin’s half-life of 19–21 days. This exogenous albumin deriving from supplemental infusion of human serum albumin and blood transfusion persists in the body for a long time, which may make liver function estimation inaccurate. Therefore, many scholars chose prealbumin as a more sensitive marker to predict prognosis than albumin during cachexia progression [27, 28]. Additionally, low preoperative prealbumin levels are a negative independent prognostic factor for cancer-specific recurrence and survival [19, 29].

A number of studies have indicated the predictive values of the neutrophil to lymphocyte ratio (NLR), platelet to lymphocyte ratio (PLR), NLR-PLR, albumin-bilirubin index (ALBI), and improved platelet-albumin-bilirubin index (PALBI). I think it may be explained as follows. First, neutrophilia inhibits the cytolytic activity of immune cells [30]. And the platelets secrete vascular endothelial growth factor and platelet-derived growth factor, which contribute to angiogenesis, cell proliferation and tumor metastasis [31]. Second, the infusion of exogenous albumin may inhabit HCC cells growth via the modulation of AFP and growth-controlling kinases [32]. And elevated serum TBIL is a sensitive marker of liver injury. A systematic review summarized serum ALB and TBIL as the two most prominent prognostic markers [33]. A recent study reported that the AUC value of NLR-PLR for predicting the 2-year OS rate was 0.653, while another study demonstrated that the AUC values of ALBI and PALBI were 0.642 and 0.675, respectively [34, 35]. The results of the present study suggested that the AUC value of the GPR-related combined indicator for predicting OS was 0.704. These indicators are different from genes or proteins, which are directly associated with the occurrence and progression of HCC, so AUC value of these markers may be not high. Through preoperative evaluation, patients are divided into different groups according to risk of recurrence and death. For patients with high risk of recurrence, follow-up strategies should be adjusted to more closely monitor the tumor progression, which help doctors take timely interferential measures to reduce the recurrence rate and improve the long-term prognosis of patients. Therefore, the GPR-related indicators for HCC patients who received combined therapies might be viewed as significant markers to effectively predict the risk of early recurrence and mortality.

Medical imaging examinations, such as CT and MRI, are not appropriate routine methods for monitoring prognosis because of the unclear visualization of microscopic metastases, high cost of inspection, and radiation hazards. Consequently, valid serum markers from routine blood and liver function examinations are of great significance for determining the optimal therapeutic modalities. However, this is a retrospective single-center study that was affected by confounding factors; thus, our results need to be validated by further multicenter studies.

## Conclusions

In summary, the proposed Fib-GPR and N-GPR scores based on independent risk factors might effectively predict the risk of early recurrence and mortality for HCC patients who received combined therapies and may possess a number of advantages for making a true clinical decision for patients with HCC who underwent TACE plus locoregional ablative therapy.

## Data Availability

The data used to support the findings are available from the corresponding author upon request.
